# Inferior immunogenicity and efficacy of respiratory syncytial virus fusion protein-based subunit vaccine candidates in aged versus young mice

**DOI:** 10.1371/journal.pone.0188708

**Published:** 2017-11-28

**Authors:** Corinne Cayatte, Angie Snell Bennett, Gaurav Manohar Rajani, Leigh Hostetler, Sean K. Maynard, Michelle Lazzaro, Patrick McTamney, Kuishu Ren, Terrence O’Day, Michael P. McCarthy, Kirsten Schneider-Ohrum

**Affiliations:** 1 Department of Infectious Diseases/Vaccines, MedImmune, Gaithersburg, Maryland, United States of America; 2 Laboratory Animal Resources, MedImmune, Gaithersburg, Maryland, United States of America; 3 Department of Statistical Sciences, MedImmune, Gaithersburg, Maryland, United States of America; Imperial College London, UNITED KINGDOM

## Abstract

Respiratory syncytial virus (RSV) is recognized as an important cause of lower and upper respiratory tract infections in older adults, and a successful vaccine would substantially lower morbidity and mortality in this age group. Recently, two vaccine candidates based on soluble purified glycoprotein F (RSV F), either alone or adjuvanted with glucopyranosyl lipid A formulated in a stable emulsion (GLA-SE), failed to reach their primary endpoints in clinical efficacy studies, despite demonstrating the desired immunogenicity profile and efficacy in young rodent models. Here, one of the RSV F vaccine candidates (post-fusion conformation, RSV post-F), and a stabilized pre-fusion form of RSV F (RSV pre-F, DS-Cav1) were evaluated in aged BALB/c mice. Humoral and cellular immunogenicity elicited after immunization of naïve, aged mice was generally lower compared to young animals. In aged mice, RSV post-F vaccination without adjuvant poorly protected the respiratory tract from virus replication, and addition of GLA-SE only improved protection in the lungs, but not in nasal turbinates. RSV pre-F induced higher neutralizing antibody titers compared to RSV post-F (as previously reported) but interestingly, RSV F-specific CD8 T cell responses were lower compared to RSV post-F responses regardless of age. The vaccines were also tested in RSV seropositive aged mice, in which both antigen forms similarly boosted neutralizing antibody titers, although GLA-SE addition boosted neutralizing activity only in RSV pre-F immunized animals. Cell-mediated immune responses in the aged mice were only slightly boosted and well below levels induced in seronegative young mice. Taken together, the findings suggest that the vaccine candidates were not able to induce a strong anti-RSV immune response in recipient mice with an aged immune system, in agreement with recent human clinical trial results. Therefore, the aged mouse model could be a useful tool to evaluate improved vaccine candidates, targeted to prevent RSV disease in older adults.

## Introduction

Respiratory Syncytial Virus (RSV) is an important cause of acute lower respiratory tract infection in young children, immunocompromised individuals, and older adults [1–3]. Most RSV infections are unrecognized in adults because patient samples are not routinely tested for RSV in this population. Recent epidemiologic studies highlight the importance of RSV as a cause of respiratory illness in adults, including illness resulting in hospitalization and death, particularly in older adults or in those with underlying cardiac or pulmonary disease [2, 4–6]. Older adults are more likely than younger adults to have severe manifestations of RSV disease [6–8], and RSV is associated with approximately 10,000 US deaths per year in persons > 65 years of age [4, 6, 9]. Despite the global RSV burden, there is to date no approved vaccine for any age group, nor is the required immune response known for a successful RSV vaccine for the older adult population.

Two surface glycoproteins, F and G, are responsible for viral entry and propagation in the host. The G protein exhibits a higher degree of variability amongst RSV strains, whereas the F protein is antigenically stable with minimal difference between subtypes [10]. For this reason, and as RSV F is the primary target for neutralizing antibodies, it has been the antigen of choice for vaccine development. However, the exact correlates of protection against lower respiratory illness complications in the elderly population remain unclear. It is well established that CD8 T cells are key players in eliminating intracellular pathogens and conferring protection against reinfection; depletion of RSV specific CD8 T cells leads to prolonged viral replication, and conversely CD8 T cell adoptive transfer enhances viral clearance [11, 12]. It has been hypothesized that enhanced RSV susceptibility in older adults is a result of immunosenescence. Immune mechanisms leading to immunosenescence are likely multifactorial, implying innate, humoral, and cellular responses. Cherukuri *et al*., showed that levels of RSV F protein-specific IFN-γ-producing T cells were lower in older individuals (65 to 85 years old) than in young adults (20 to 35 years old) [13], suggesting that deficient RSV F-specific T cell responses might increase susceptibility to severe RSV disease in the older adult population. Similarly, deficiencies in RSV-specific CD8+ cytolytic T cell responses and gamma interferon (IFNγ) responses [14, 15] have been identified in aged BALB/c mice compared to young animals, which may impair responses to RSV infection. A more detailed study focusing on quantitative and qualitative changes in RSV-specific CD8 T cells isolated from aged BALB/c mice showed a profound decrease of immunodominant M2_82-90_ and subdominant F_85-93_ and M2_127-135_ CD8 T responses in lung parenchyma and airways at the peak of T cell response 8 days post-infection [16]. Additionally, an alum adjuvanted RSV post-F subunit vaccine evaluated in aged BALB/c mice showed reduced immunogenicity compared to young mice, suggesting that subunit vaccines developed for the older adult population might require a strong adjuvant to overcome immunosenescence, to achieve protective neutralizing antibody titers, and a Th1-biased cytokine response to limit lung inflammation [15].

Post-fusion RSV F (RSV post-F) adjuvanted with the toll-like receptor-4 (TLR-4) agonist GLA formulated in SE was under development as a vaccine candidate for older adults. This vaccine candidate demonstrated promising immunogenicity and efficacy in rodent models in young animals [17–19], and boosted RSV specific humoral and cellular immune response in older adults in a Phase 1 study [17, 20]. However, this vaccine failed to prevent RSV disease in a Phase 2 clinical trial (NCT02508194). Similarly, a non-adjuvanted RSV F nanoparticle vaccine failed to prevent disease in older adults ≥ 60 years of age in a Phase 3 clinical trial (NCT02608502). Detailed analysis of RSV post-F + GLA-SE immunization has not yet been undertaken in aged rodent models and the goal of this study was to compare the immunogenicity and efficacy of RSV F +/- adjuvant vaccine candidates in young and aged mice, and to investigate (in addition to the failed RSV post-F based vaccine candidates), the potentially more potent and promising pre-fusion (pre-F) form of RSV F [21] in both RSV seronegative and seropositive aged BALB/c mice.

## Results

### Humoral and cellular immune response after immunization with post-fusion RSV F in the presence or absence of GLA-SE adjuvant in RSV seronegative young and aged mice

**Fig 1:**
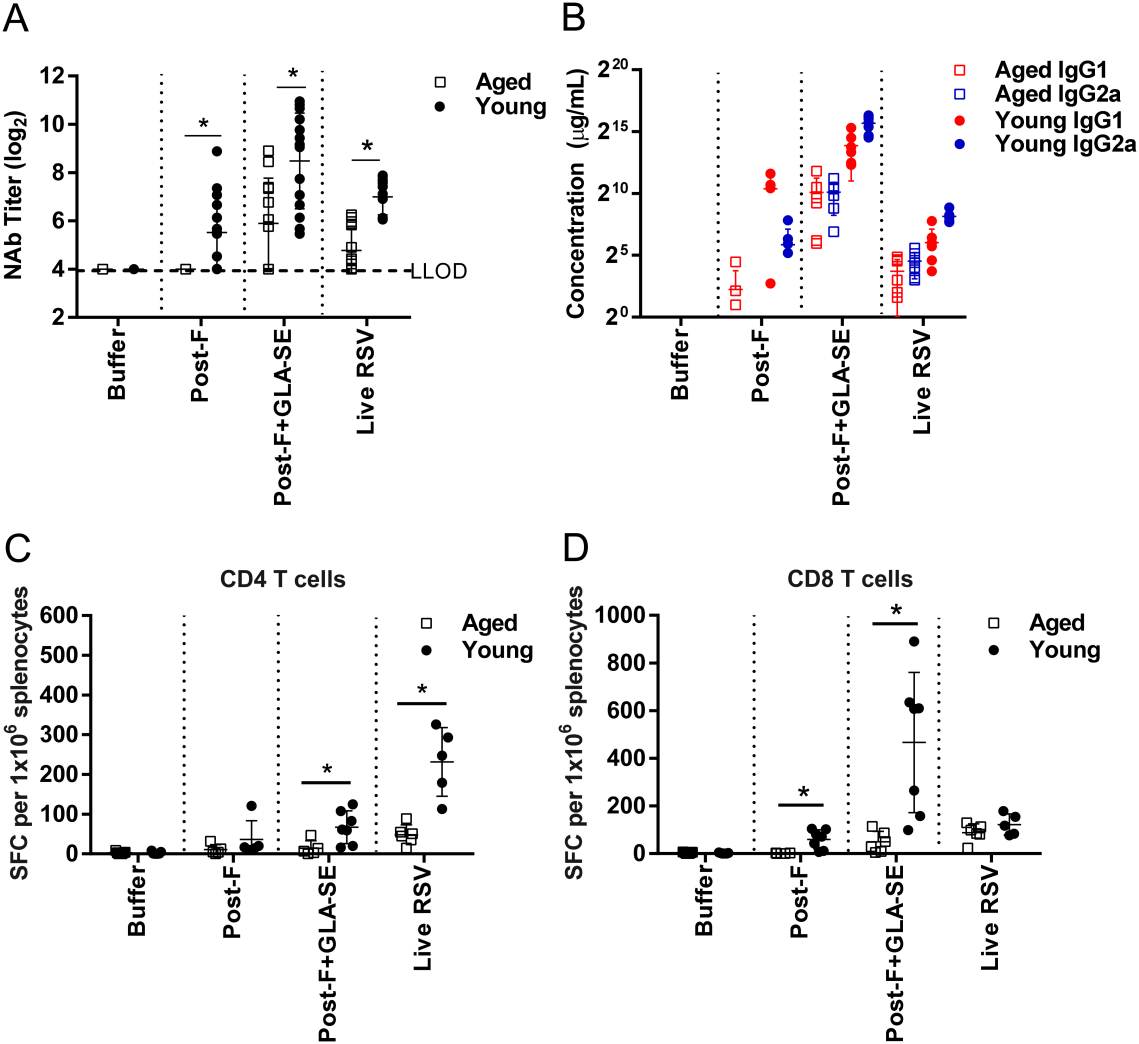
Immunogenicity of post- RSV F formulations in young and old seronegative mice. Young (7 weeks old) and aged (18 months old) BALB/c mice (n = 4 to 7 per group) were immunized i.m. at day 0 and day 21 with buffer alone, post-F (0.3 μg) +/- GLA-SE (2.5 μg/2%) adjuvant. A control group for natural infection was immunized i.n. with live RSV (10^6^ PFU) at day 0. At day 35, animals were challenged i.n. with 10^6^ PFU of wt RSV A2. (A) Prior to challenge (day 34), sera were harvested and NAb titers were evaluated using a microneutralization assay. Data is presented as the log_2_ dilution of serum that provides 50% reduction in viral entry with a LLOD of 4 indicated by a dashed line. At sacrifice (day 39), (B) RSV F specific anti-IgG1 and anti-IgG2a were measured by ELISA, and splenocytes were isolated and stimulated for 24 h with peptides representing (C) RSV F specific CD4 T-cell and (D) CD8 T cell epitopes. The number of IFNγ secreting cells per 10^6^ splenocytes was determined by ELISPOT. Group means ± SD of individual mice are shown. For statistical analyses, aged and young mice were compared. *, P<0.05.

Firstly, aged (18 months) and young (7 weeks) BALB/c mice were immunized twice, intramuscularly with 0.3 μg of RSV post-F alone or in the presence of 2.5 μg GLA in 2% stable emulsion (GLA-SE). The antigen dose was chosen based on previous experience in young mice, in which 0.3 μg of RSV post-F + GLA-SE confers complete protection from RSV replication in lower and upper respiratory tract [18]. RSV post-F alone induced measurable neutralizing antibody titers in 8 out of 14 young mice (mean titer 5.5log_2_) but no detectable neutralizing antibodies in the aged animals (Fig 1A). Addition of GLA-SE increased the neutralizing antibody titers in the young animals (mean titer 9.5log_2_) and induced neutralizing activity in 7 out of 12 aged mice, albeit at statistically significant lower levels than the titers induced in young mice (mean titer 5.9log_2_). Live RSV A2 infection induced neutralizing antibody titers, which were also significantly lower in the aged mice (mean titer 4.8log_2_) compared to young mice (mean titer 7.0log_2_).

To evaluate T helper 1 and T helper 2 (Th1/Th2) type responses to the vaccine formulations, we determined the isotypes of the RSV F-specific antibodies elicited after immunization. IFNγ promotes class-switching of antibodies from IgG1 to IgG2a in the mouse, making IgG2a antibodies a good correlate of Th1-biasing IFNγ. RSV post-F immunization without adjuvant induced an IgG1 biased RSV F specific antibody response in the young mice, suggesting a Th2 skewed immune response, whereas the antibody response in the aged mice was lower for IgG1 and below the limit of detection for IgG2a (Fig 1B). Addition of GLA-SE adjuvant to RSV post-F elicited a balanced IgG1/IgG2a response in the aged mice and IgG2a biased antibody response in the young mice (Fig 1B).

**Fig 2:**
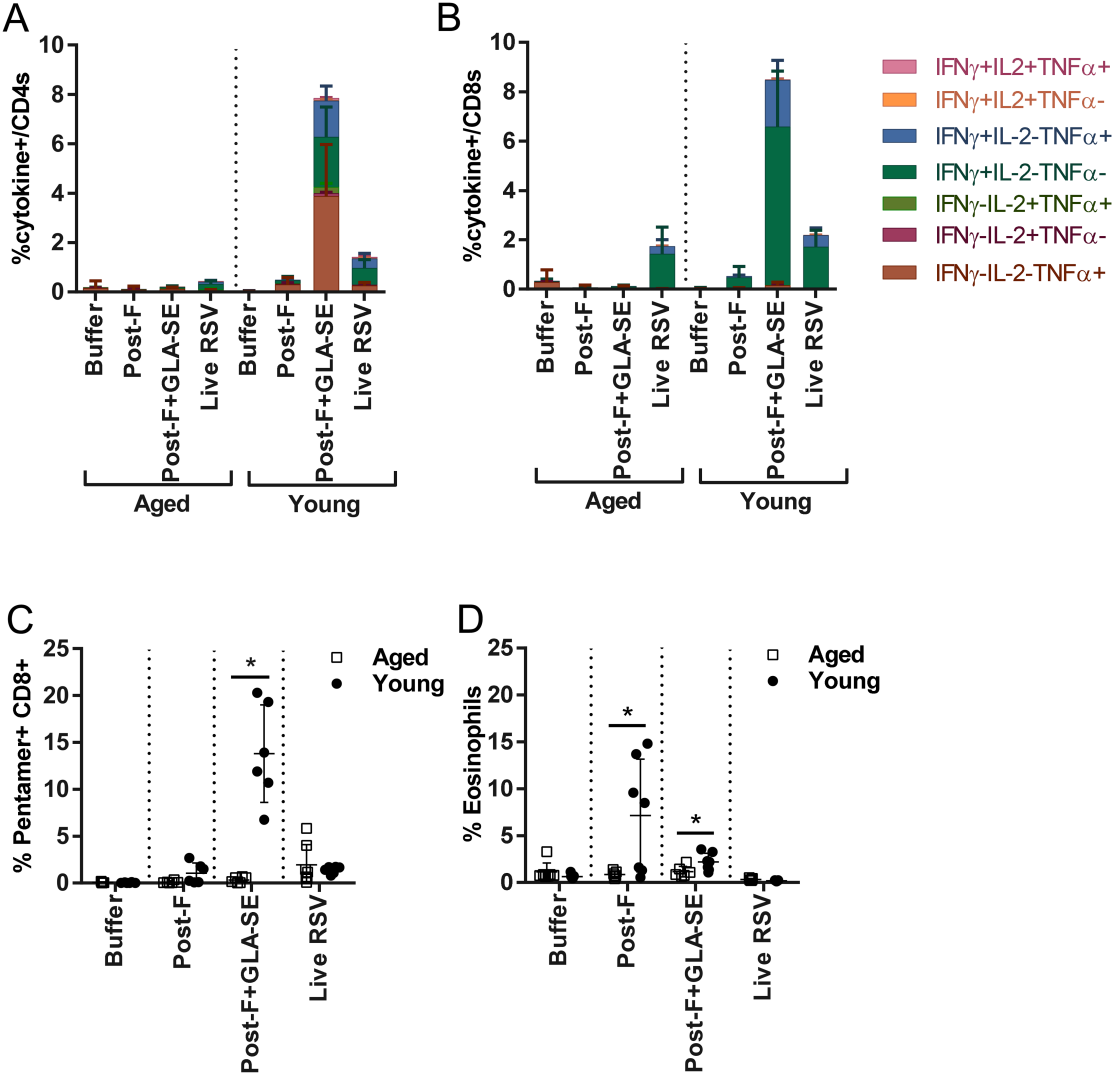
Lung T cell profile after RSV challenge in young and aged seronegative mice immunized with post-RSV F formulations. Young (7 weeks old) and aged (18 months old) BALB/c mice (n = 4 to 7 per group) were immunized i.m. at day 0 and day 21 with buffer alone, post-F (0.3 μg) +/- GLA-SE (2.5 μg/2%) adjuvant. A control group for natural infection was immunized i.n. with live RSV (10^6^ PFU) at day 0. At day 35, animals were challenged i.n. with 10^6^ PFU of wt RSV A2. 4 days post-challenge (day 39), lung cells were isolated and stimulated with RSV F peptide pool to evaluate intracellular cytokine expression by flow cytometry. Cells were surface stained for CD3 and CD8, intracellularly stained for IFNγ, TNFα, and IL-2, and analyzed on an LSR II for the frequency of responding (A) CD4+ and (B) CD8+ T cells. (C) The percentage of F85-93-pentamer+ CD8 T cells was determined by flow cytometry. (D) The percentage of lung eosinophils was determined by flow cytometry. For (C) and (D), the mean of individual values +/- SD is shown. For statistical analyses, aged and young mice were compared. *, P<0.05.

The cell-mediated immune response to RSV is decreased in older adults [13] and a successful RSV vaccine for the older population may need to boost RSV-specific T cell responses. We therefore compared the induction of RSV F specific CD4 and CD8 T cells in the spleens of aged and young mice after RSV post-F or RSV post-F + GLA-SE vaccination via IFNγ ELISPOT analysis (Fig 1C and 1D). Additionally, we conducted flow cytometric analysis of lung lymphocytes in immunized and subsequently RSV A2-challenged mice (Fig 2), focusing on Th1 cytokine responses as it has been shown previously that RSV sF + GLA-SE vaccine primes IFNγ-producing T cell responses in multiple rodent models [18]. Splenic RSV F-specific CD4 T cells secreting IFNγ (I-E^d^, GWYTSVITIELSNIKE epitope) were very low or undetectable in young and aged mice after RSV post-F immunization, respectively and slightly higher levels were induced only in the young mice when RSV post-F was administered with GLA-SE (Fig 1C). Similarly, CD8 T cell IFNγ responses directed to the RSV F (H2-K^d^) F_85-93_ KYKNAVTEL epitope in the aged mice were undetectable in the absence, and very low in the presence, of adjuvant. In contrast, in the young animals the CD8 T cell response was markedly increased in the presence of adjuvant (with and without adjuvant, SFC means/1x10^6^ cells 60 and 466.3, respectively) (Fig 1D). Live RSV A2 infection induced substantially lower levels of RSV F specific CD4 T cells in the spleens of aged mice, compared to young mice (mean SFC/1x10^6^ cells 48.3 and 231.6, respectively), whereas the RSV F specific CD8 T cell response was comparable (Fig 1C and 1D). To characterize the potential localization of vaccine-induced CD4 and CD8 T cells to the site of viral replication, lung lymphocytes were harvested at day 4 post RSV A2 challenge for flow cytometric analysis of functional cytokine responses (IFNγ, TNFα, and IL-2) to RSV F overlapping peptide pool restimulation. Immunization with post-F alone did not induce RSV F specific, cytokine secreting CD4 or CD8 T cells above buffer controls in either age group (Fig 2A and 2B) consistent with the observations made with splenic lymphocytes. Likewise, in the aged mice vaccination with RSV post-F with GLA-SE adjuvant did not result in recruitment of RSV F-specific, functional CD4 or CD8 T cells to the lungs (Fig 2A and 2B). In contrast, RSV post-F + GLA-SE immunization of young mice did result in recruitment of RSV F specific, functional CD4 and CD8 T cells (mean of 7.8% and 8.5% respectively) (Fig 2A and 2B). These RSV F specific CD4 T cells, were IFNγ/TNFα dual positive (1.5%), IFNγ single positive (2%), and TNFα single positive (3.9%) (Fig 2A). In contrast, RSV F specific CD8 T cells were primarily IFNγ/TNFα dual positive (1.9%) or IFNγ single positive (6.4%) and no TNFα single positive CD8 T cells were detectable (Fig 2B). Staining of lung lymphocytes with a CD8 Pentamer specific for the (H2-K^d^) F_85-93_ epitope confirmed the above findings (Fig 2C). Given the Th2-biased antibody response in animals immunized with RSV post-F alone (Fig 1B) we investigated the influx of eosinophils into the lungs of vaccinated and RSV-challenged young and aged mice (Fig 2D). As previously observed by histological examination [15], only the RSV post-F immunized, young animals demonstrated an influx of eosinophils into their lungs after virus challenge, whereas no significant eosinophil influx above buffer control was observed in any of the groups of the aged mice cohort.

### Efficacy of GLA-SE adjuvanted RSV post-F in young and aged mice at conferring protection from RSV challenge

**Fig 3:**
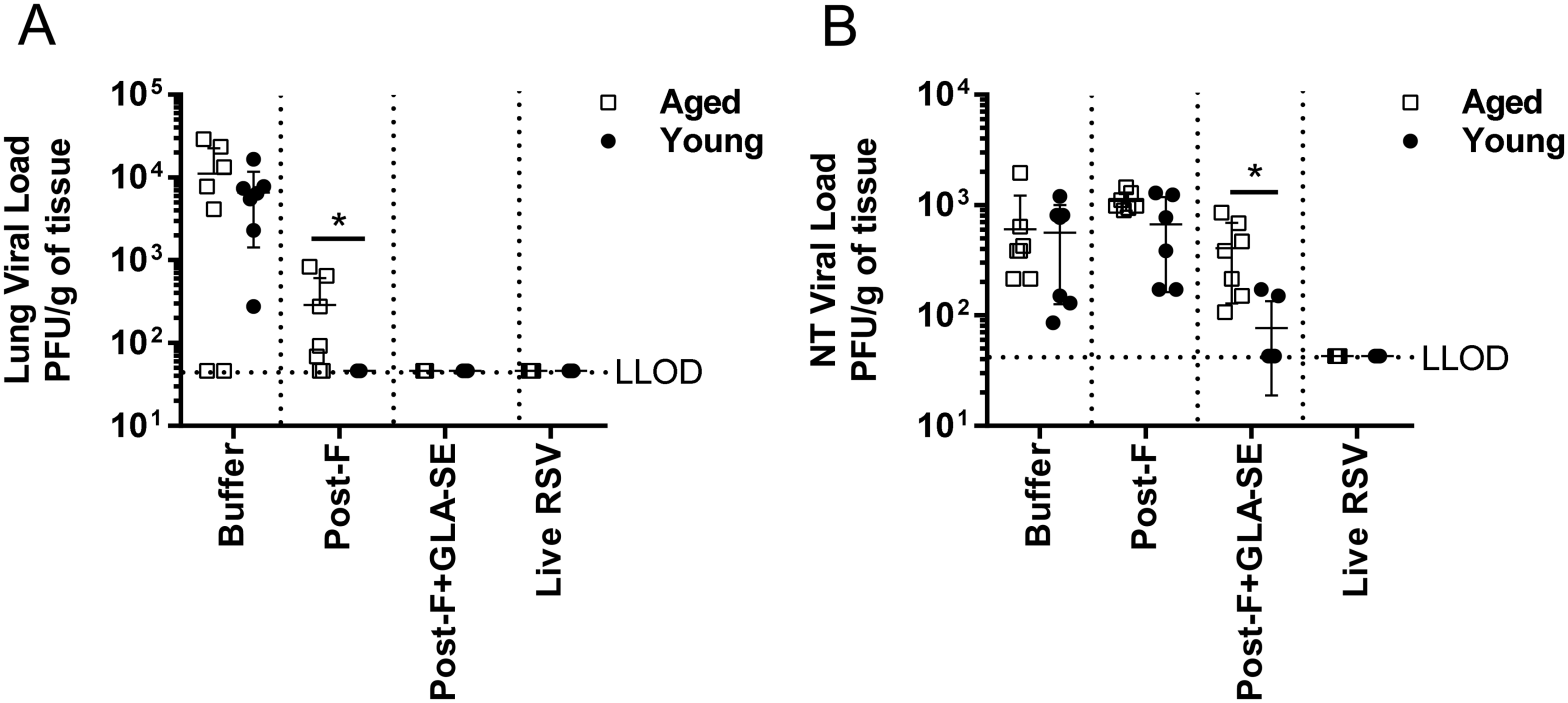
Viral titers post RSV challenge in young and old seronegative mice immunized with post-RSV +/- GLA-SE. Young (7 weeks old) and aged (18 months old) BALB/c mice (n = 4 to 7 per group) were immunized i.m. at day 0 and day 21 with buffer alone, post-F (0.3 μg) +/- GLA-SE (2.5 μg/2%) adjuvant. A control group for natural infection was immunized i.n. with live RSV (10^6^ PFU) at day 0. At day 35, animals were challenged i.n. with 10^6^ PFU of wt RSV A2. 4 days post-challenge (day 39), (A) lung and (B) nasal turbinates viral loads were determined by plaque assay. The mean of individual values +/- SD is shown. For statistical analyses, aged and young mice were compared. *, P<0.05.

Next, the GLA-SE adjuvanted and non-adjuvanted RSV post-F vaccine candidates were evaluated for their ability to protect immunized young and aged mice from RSV A2 virus challenge, by determining the virus titers in their lungs and nasal turbinates 4 days post intranasal administration. The mice immunized with protein buffer alone or primed with RSV A2 served as negative and positive controls, respectively (Fig 3A and 3B). As expected, prior infection with RSV A2 resulted in complete protection from secondary RSV infection in upper and lower respiratory tracts of young and aged mice (Fig 3A and 3B). RSV post-F immunization without the addition of adjuvant completely protected young mice from virus replication in their lungs. In the aged mice the lung virus titers were reduced by 1.4 log_10_ in animals that received RSV post-F without adjuvant compared to buffer immunized mice (Fig 3A), despite the lack of detectable serum neutralizing antibody activity and T cell response (Fig 1A and 1B). RSV post-F + GLA-SE did confer complete protection from virus replication in the lungs of young and aged mice (Fig 3A). Viral titers were also assessed in nasal turbinates, where inducing protection can be more challenging than in the lungs [22]. Consistent with the lower neutralizing antibody titers and very low RSV F specific T cell responses elicited in the aged mice after RSV post-F immunization, even in the presence of GLA-SE adjuvant, virus titers were not reduced in the nasal turbinates of aged mice immunized with RSV post-F alone and only slightly reduced in the animals that received RSV post-F + GLA-SE, compared to mice immunized with buffer alone (Fig 3B). Similarly, in the young mice, immunization with RSV post-F alone did not reduce virus replication in the upper respiratory tract. Viral titers in the upper airways of young mice vaccinated with GLA-SE-adjuvanted post-F were lower compared to those in similarly vaccinated aged mice, providing complete protection in 3 out of 5 young animals (Fig 3B).

### RSV pre-F + GLA-SE induces lower levels of RSV F specific CD8 T cells compared to RSV post-F in young mice

**Fig 4:**
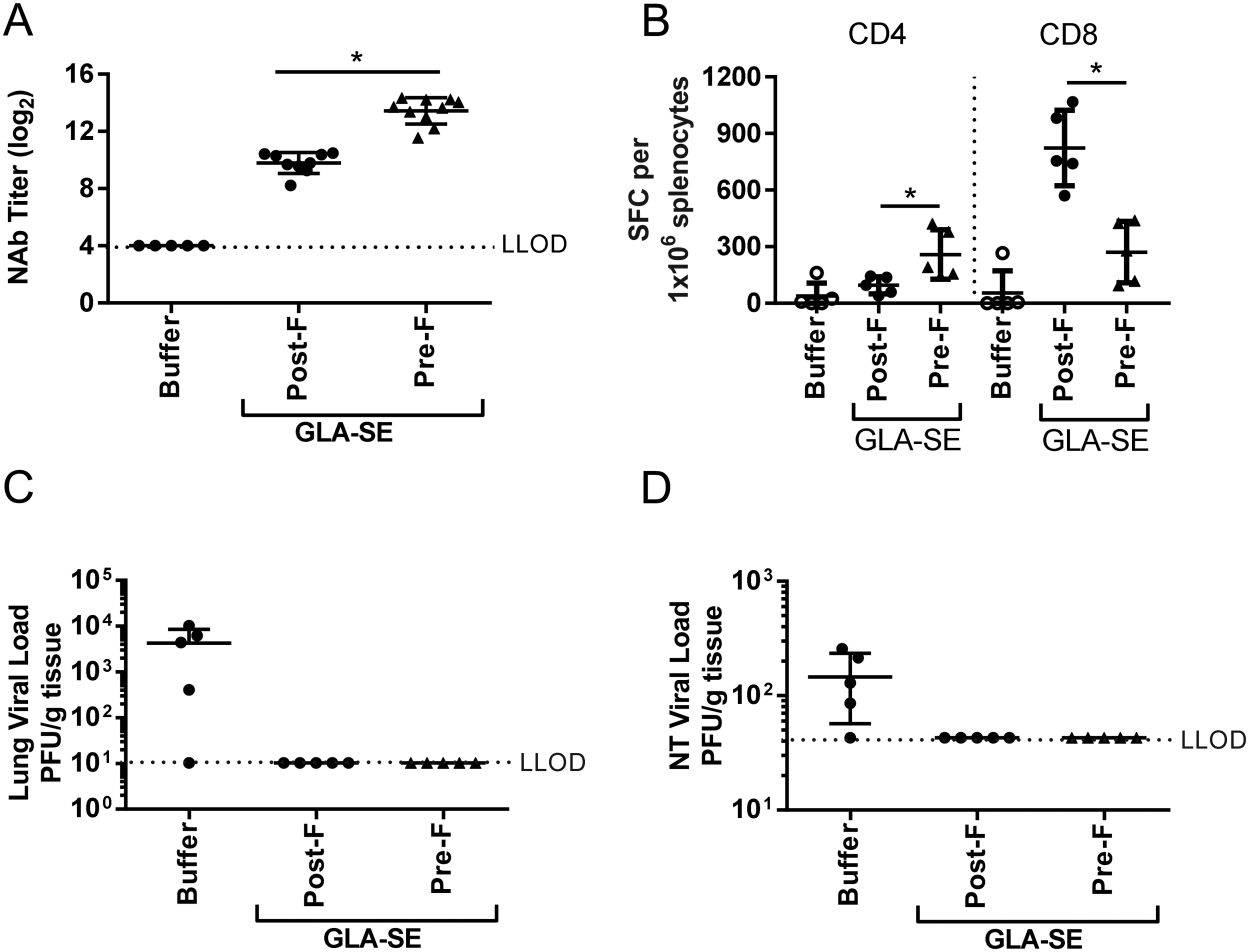
Immunogenicity and protection from viral challenge of pre-RSV + GLA-SE in young, seronegative mice. Young (7 weeks old) BALB/c mice (n = 5 to 10 per group) were immunized i.m. at day 0 and day 21 with buffer alone, RSV F pre- and post-conformation at 0.3 μg +/- GLA-SE (2.5 μg/2%) adjuvant. At day 35, animals were challenged i.n. with 10^6^ PFU of wt RSV A2. (A) Prior to challenge (day 34), sera were harvested and NAb titers were evaluated using a microneutralization assay. Data is presented as the log_2_ dilution of serum that provides 50% reduction in viral entry with a LLOD of 4 indicated by a dashed line. (B) 4 days post-challenge (day 39), splenocytes were isolated and stimulated for 24 h with RSV F specific CD4 T-cell and CD8 T cell epitopes. The number of IFNγ secreting cells per 10^6^ splenocytes was determined by ELISPOT. (C) Lung and (D) nasal turbinates viral loads were measured by plaque assay. Group means ± SD of individual mice are shown. For statistical analyses, aged and young mice were compared. *, P<0.05.

A stabilized, soluble form of RSV F in the pre-fusion (RSV pre-F) conformation has been demonstrated to induce higher levels of neutralizing antibodies than soluble RSV post-F in mice, cotton rats and non-human primates, and might serve as a preferred antigen for a RSV subunit vaccine [21, 23, 24]. To date, only limited data is available on the induction of RSV F specific T cells after RSV pre-F immunization. Therefore, prior to evaluating immune responses to pre-fusion F in aged mice, we tested the immune responses and protective efficacy induced by this antigenic form of RSV F, in young mice. As observed previously with other adjuvants in mice and non-human primates (and in the context of GLA-SE in cotton rats) RSV pre-F + GLA-SE did induce 14.5 fold higher neutralizing antibody titers than post-F + GLA-SE (Fig 4A). Surprisingly, this trend did not hold true for the induction of RSV F specific T cell responses, as RSV pre-F induced statistically significant reduction in CD8 T cells secreting IFNγ compared to RSV post-F immunization (mean of 271 vs. 823 SFC/1x10^6^ splenocytes, respectively) and higher CD4 T cells (mean of 259 vs. 95 SFC/1x10^6^ splenocytes, respectively) (Fig 4B). Both RSV post-F and RSV pre-F in combination with GLA-SE conferred complete protection from RSV A2 challenge in upper and lower respiratory tract in young mice (Fig 4C and 4D).

### Immunogenicity and efficacy of RSV post-F and pre-F in aged, seronegative mice

**Fig 5:**
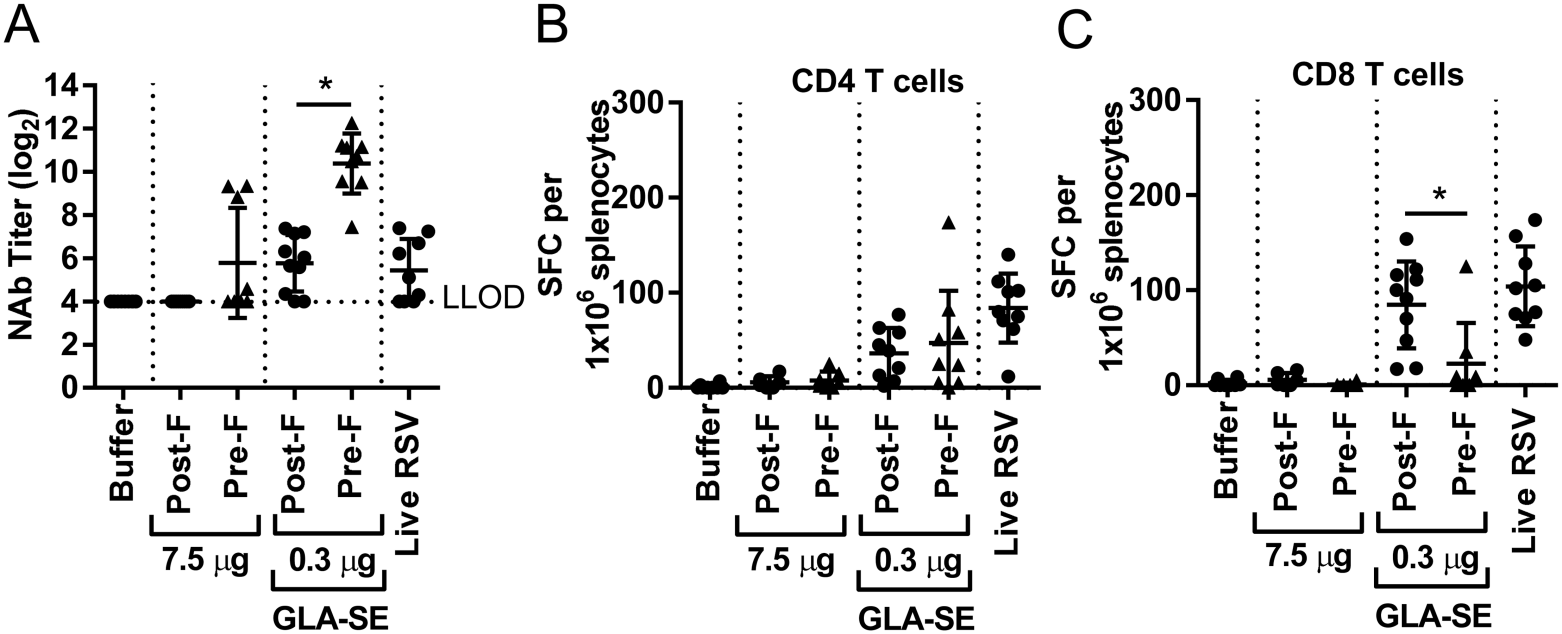
Immunogenicity of post-F and pre-F in aged seronegative mice. Aged (18 months old) BALB/c mice (n = 6 to 10 per group) were immunized i.m. at day 0 and day 21 with buffer alone, RSV F (pre- or post- conformation) at the indicated doses +/- GLA-SE (2.5 μg/2%) adjuvant. A control group for natural infection was immunized i.n. with live RSV (10^6^ PFU) at day 0. At day 35, animals were challenged i.n. with 10^6^ PFU of wt RSV A2. Prior to challenge (day 34), sera were harvested and NAb titers were evaluated using a microneutralization assay. Data is presented as the log_2_ dilution of serum that provides 50% reduction in viral entry with a LLOD of 4 indicated by a dashed line. 4 days post-RSV A2 challenge, splenocytes were isolated and stimulated for 24 h with peptides representing **(B)** RSV F specific CD4 T cell and **(C)** CD8 T cell epitopes. The number of IFNγ secreting cells per 10^6^ splenocytes was determined by ELISPOT. Group means ± SD of individual mice are shown. For statistical analyses, aged and young mice were compared. *, P<0.05.

Given the superior induction of neutralizing antibodies by RSV pre-F in young mice, we compared the capacity of RSV pre-F and post-F to induce neutralizing antibodies and T cells in aged mice. Similar to the young mice, RSV pre-F induced higher levels of neutralizing antibodies than RSV post-F in the aged mice, in the absence (mean titer 5.8 log_2_ vs. no detectable titer above the LOD of 4) or presence of GLA-SE adjuvant (mean titer 10.4log_2_ vs. 5.7log_2_) (Fig 5A). After challenge with live RSV A2 at day 35, spleens were harvested for T cell response analysis, and lungs and nasal turbinates were used to assess protection from virus replication. Neither antigen induced detectable RSV F specific CD4 or CD8 T cells above buffer control in the absence of GLA-SE (Fig 5B and 5C). In the presence of adjuvant, RSV post-F and pre-F induced similar, low numbers of RSV F specific CD4 T cells (Fig 5B), whereas the induction of RSV F specific CD8 T cells was significantly higher (comparable to live virus immunization) when the mice were immunized with RSV post-F compared to pre-F (Fig 5C).

**Fig 6:**
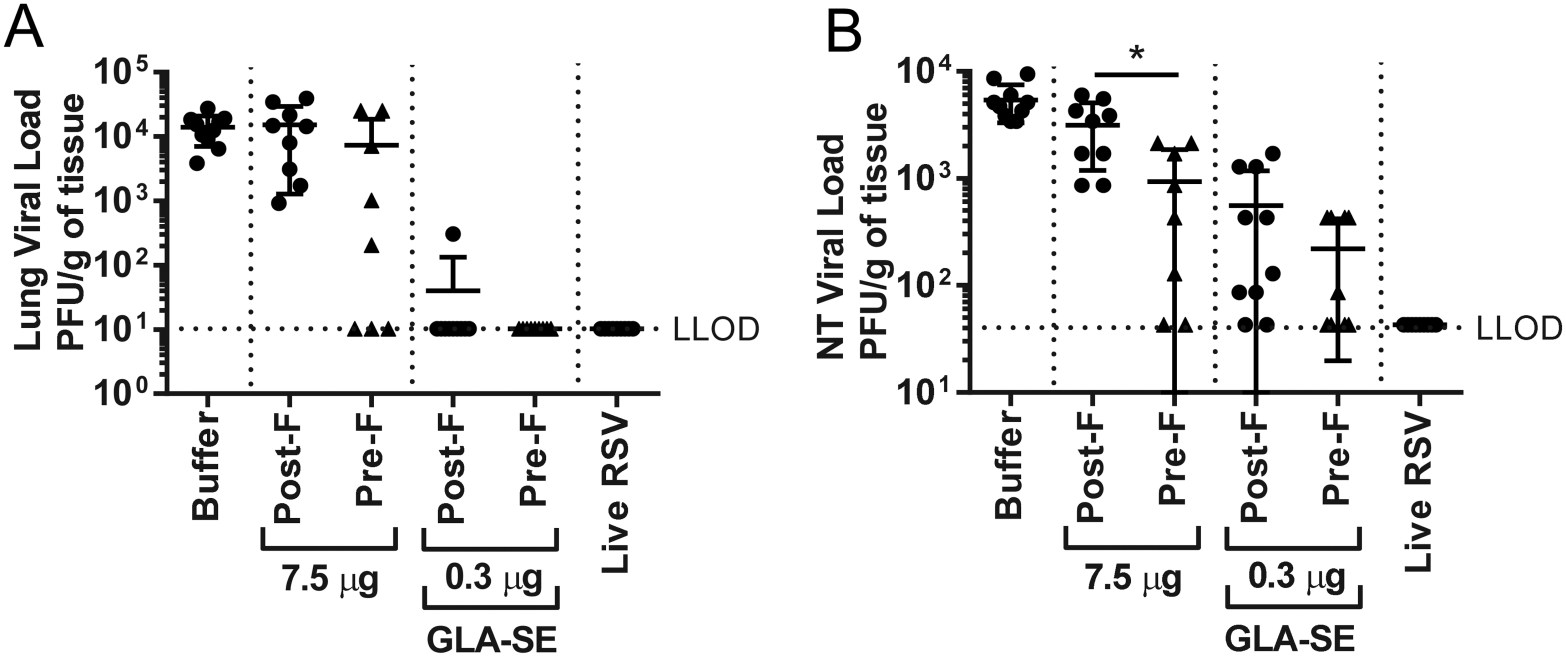
Protection from RSV A2 challenge in aged seronegative mice, immunized with pre- and post-RSV F. Aged (18 months old) BALB/c mice (n = 6 to 10 per group) were immunized i.m. at day 0 and day 21 with buffer alone, RSV F (pre- or post- conformation) at the indicated doses +/- GLA-SE (2.5 μg/2%) adjuvant. A control group for natural infection was immunized i.n. with live RSV (10^6^ PFU) at day 0. At day 35, animals were challenged i.n. with 10^6^ PFU of wt RSV A2. 4 days post-RSV A2 challenge, **(A)** lung and **(B)** nasal turbinates viral loads were measured by plaque assay. For statistical analyses, aged and young mice were compared. *, P<0.05.

RSV post-F immunization without the addition of GLA-SE did not confer protection from virus replication in the lungs or nasal turbinates, while RSV pre-F completely protected the lungs of 3 out of 8 animals and prevented virus replication in the nasal turbinates in 2 out of 8 mice, even in the absence of adjuvant (Fig 6A and 6B). Addition of GLA-SE to RSV post-F vaccination resulted in protection of 9 out of 10 aged mice from RSV replication in their lungs. Likewise, RSV pre-F + GLA-SE immunization completely abrogated detectable virus replication in the lower respiratory tract (Fig 6A). In the upper respiratory tract, RSV post-F + GLA-SE completely protected 2 out of 8 mice and reduced the mean virus titer by 1.3 log_10_ compared to buffer immunized animals (Fig 6B). RSV pre-F immunization reduced the virus titer even further and completely protected 4 out of 9 animals (Fig 6B).

### RSV post-F and pre-F with or without GLA-SE boosts pre-existing neutralizing activity in seropositive aged mice

**Fig 7:**
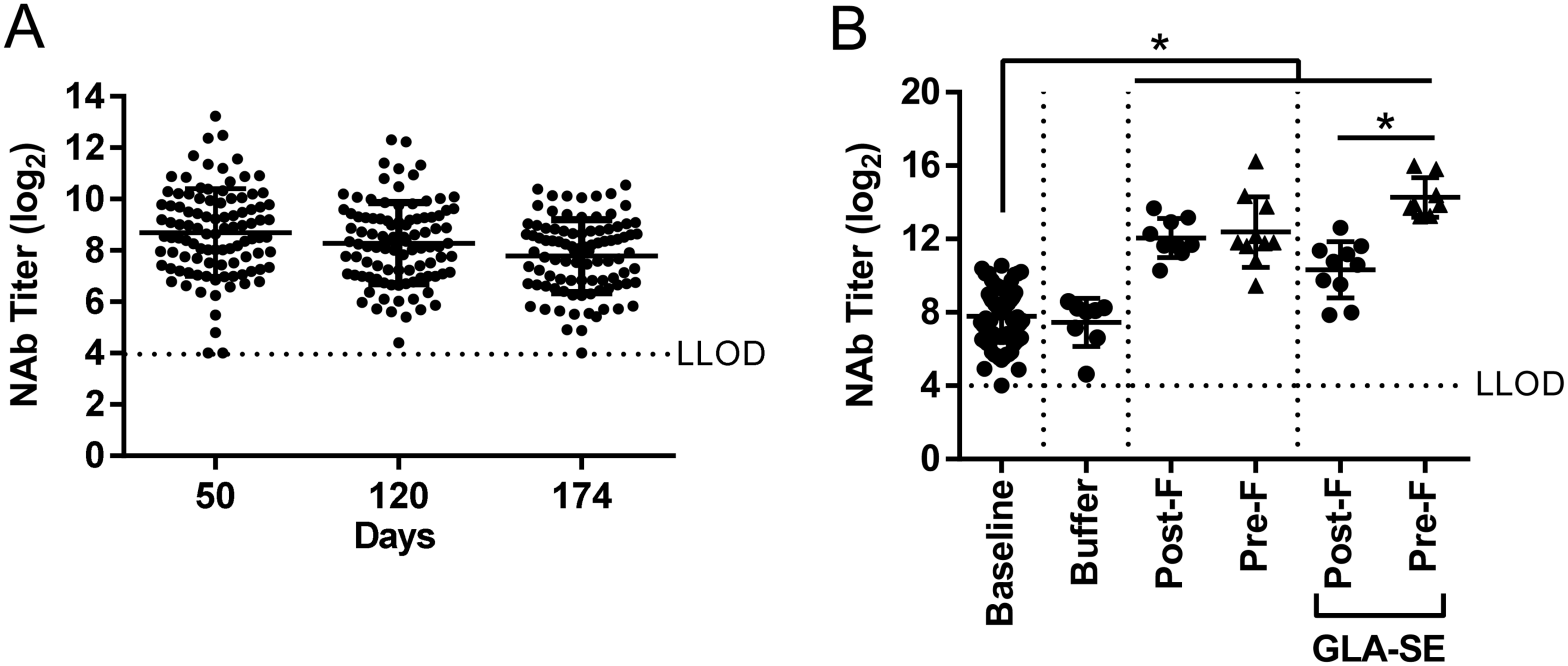
NAb induction of pre-F and post-F formulations in aged seropositive mice. 12 months old mice were i.n. immunized with RSV A2 (10^6^ PFU) at day 0 and RSV B (10^5^ PFU) at day 60. Aged seropositive mice were then immunized i.m. at day 176 (18 months old) with buffer alone, post-F or pre-F (0.3 μg) +/- GLA-SE (2.5 μg/2%) adjuvant. **(A)** Bleeds were collected at day 50, 120, and 174 to assess the levels of RSV A2 neutralizing antibodies in seropositive animals prior to immunization (baseline). Data is presented as the log_2_ dilution of serum that provides 50% reduction in viral entry with a LLOD of 4 indicated by a dashed line. **(B)** 14 days post immunization, RSV A2 neutralization titers were evaluated. Group means ± SD of individual mice are shown. For statistical analyses, multiplicity adjusted ANOVA test was performed against baseline as a control group, followed by pairwise testing of post-F versus pre-F, * for P<0.05.

Older adults are naturally RSV seropositive and RSV vaccines for older adults are developed with the goal of boosting pre-existing immunity. Therefore we investigated a boost immunization in RSV seropositive, aged mice. Twelve month old BALB/c mice were infected with RSV A2 and then to mimic natural infection patterns found in humans more closely (exposure to several RSV strains throughout the lifetime), the animals were infected with RSV B 60 days later. At day 176 post RSV A2 infection (18 months of age) the aged mice were immunized with buffer alone or RSV post-F and pre-F plus or minus GLA-SE adjuvant. Serum was collected at day 50, day 120 and day 174 post RSV A2 infection to assess the neutralization antibody titers induced by live RSV exposures, which served as the baseline titer (Fig 7A). A single RSV post-F or pre-F immunization without GLA-SE boosted the neutralizing antibody titers (16.8 fold and 18.4 fold, respectively) compared to baseline (day 174, RSV A2 neutralizing titer); RSV pre-F was not superior at boosting pre-existing neutralizing antibodies (Fig 7B). RSV post-F boosted the neutralizing antibody titers to a similar extent, whether adjuvant was present or not. The addition of GLA-SE resulted in a statistically significant improvement in the boosting effect conferred by RSV pre-F (26 fold over baseline vs. 18.4 fold over baseline without adjuvant) (Fig 7B).

**Fig 8:**
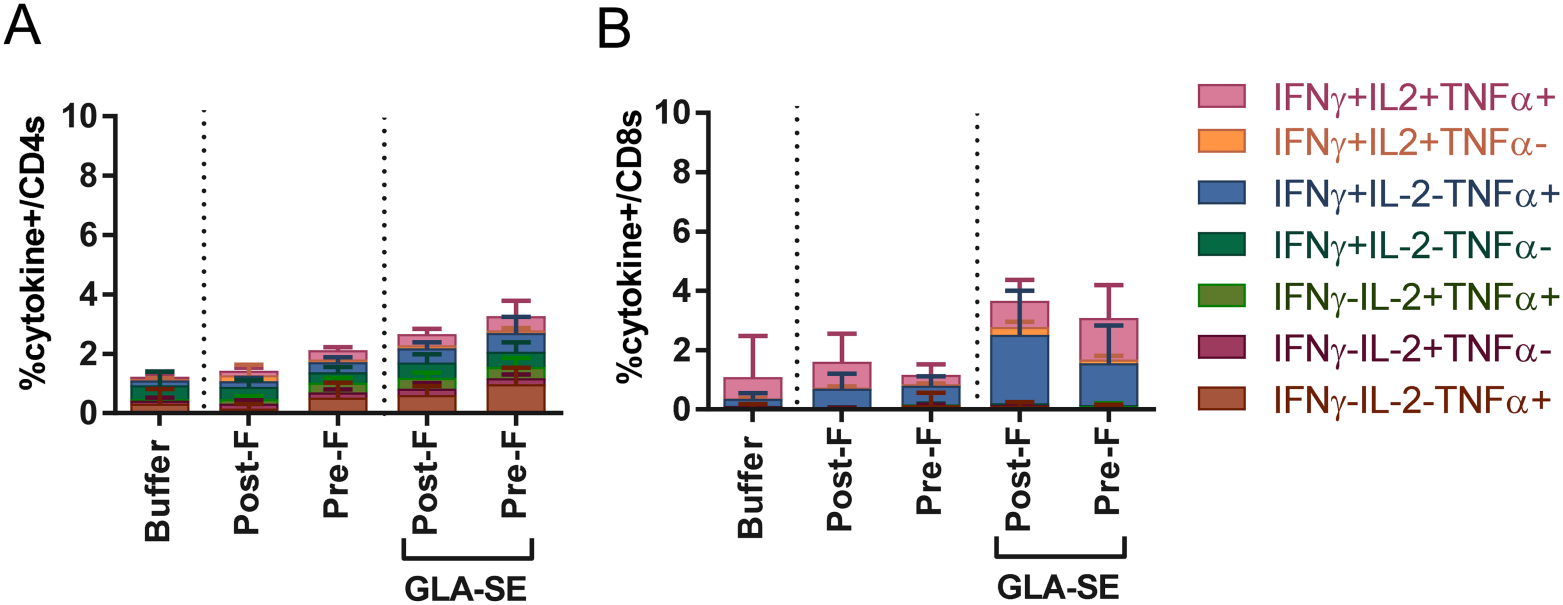
Lung T cell profile of aged seropositive mice following immunization with different RSV F formulations and post challenge with RSV A2. 12 months old mice were i.n. immunized with RSV A2 (10^6^ PFU) at day 0 and RSV B (10^5^ PFU) at day 60. Aged seropositive mice were then immunized i.m. at day 176 (18 months old) with buffer alone, post-F or pre-F (0.3 μg) +/- GLA-SE (2.5 μg/2%) adjuvant. At day 190, aged seropositive mice were challenged with RSV A2 (10^6^ PFU). 4 days later, lung cells were isolated and stimulated with RSV F peptide pool. Cells were then surface stained for CD3 and CD8, intracellularly stained for IFNγ, TNFα, and IL-2, and analyzed on an LSR II for the frequency of responding **(A)** CD4+ and **(B)** CD8+ T cells.

As boosting of pre-existing T cell responses and the recruitment of T cells to the lung after RSV exposure may be important for a successful RSV vaccine for older adults, the next step was to determine whether RSV post-F and pre-F immunization boosts pre-existing T cells which were measured in the lungs 4 days after RSV A2 challenge. The percentages of RSV F specific CD4 and CD8 T cells were determined by intracellular cytokine staining and flow cytometry (Fig 8A and 8B). RSV post-F immunization alone, did not induce significant recruitment of RSV F specific CD4 T cells (1.4% vs. 1.1% for buffer alone), and the addition of GLA-SE moderately boosted the CD4 T cell levels to 2.7%. A higher impact on CD4 T cell recruitment was noted with RSV pre-F, with an increase to 2.1% without adjuvant, and to 3.2% with adjuvant. On the other hand, the levels of CD8 T cells were not statistically boosted by unadjuvanted RSV post-F or pre-F, whereas GLA-SE addition resulted in similar boosting and recruitment of pre-existing CD8 T cells (3.6% and 3.1% respectively).

## Discussion

To date, most preclinical studies related to the development of RSV vaccines for older adults have been performed in young, RSV seronegative rodent models and have demonstrated the potential for RSV F antigen in a pre- or post-conformation to induce a strong and protective immune response, especially in the presence of an adjuvant [18, 21, 23, 25]. Cherukuri et al., evaluated the efficacy of RSV post-F and showed impaired F-specific antibody responses, neutralization titers, and induction of lung cytokines after viral challenge of aged mice compared to young animals. They also demonstrated that the addition of alum effectively increased the neutralizing antibody titers, but had no impact on lung cytokine activation post-challenge, failing to fully prevent lung viral replication, perhaps due to the lack of an efficient CD4 and/or CD8 T cell response [15]. Here, we demonstrated in RSV seronegative aged mice that addition of GLA-SE to RSV post-F induced higher neutralization titers than antigen alone, fully protected the lungs from viral replication, but only modestly induced F specific CD4 and CD8 T cell responses, which may explain the lack of protection from viral replication in the upper respiratory tract post challenge (Figs 1, 2 and 3). The same, very low induction of CD4 and CD8 T cells was observed in aged mice vaccinated with RSV pre-F + GLA-SE, albeit with higher neutralizing antibody titers observed after immunization with the antigen in the pre-fusion conformation compared to immunization with RSV post-F (Fig 5). Interestingly, even though the amino acid sequence of the immunodominant RSV F specific CD4 and CD8 T cell epitopes are identical in the RSV pre-F and post-F antigens used in this study, RSV pre-F induced higher CD4 but lower CD8 T cell responses compared to RSV post-F in the young mice and lower CD8 T cell responses in RSV naïve aged mice (Figs 4B and 5C). This difference is not apparent in the seropositive aged mice boosted with RSV pre-F and post-F (Fig 8). Whether this finding is a model-related artifact or due to differences in antigen processing and presentation remains unclear. However, consistent with this impairment in CD8 T cell induction, RSV pre-F failed to fully protect aged mice from viral replication in the upper respiratory tract (Fig 6). This is in strong contrast to the efficacy obtained in young animals in which RSV post-F and pre-F in the presence of GLA-SE offered complete protection from virus replication in the lower and upper respiratory tract (Fig 4). Of note, only the RSV A2 pre-exposed, aged control animals (despite low to undetectable circulating neutralization titers) were able to control virus in both lungs and nasal turbinates. This might be due to an enhanced ability of the live virus, administered intranasally, to generate protective mucosal immune responses, compared to the subunit vaccine candidates that were evaluated. In summary, neither RSV post-F nor pre-F adjuvanted with GLA-SE were able to induce a completely protective immune response (upper and lower respiratory tract) in aged RSV seronegative mice, at the evaluated vaccine dose.

Next we investigated unadjuvanted and adjuvanted RSV F antigens in an even more relevant model, by pre-exposing mice to a RSV A and B infection during the aging process. We showed that RSV pre-F + GLA-SE but not unadjuvanted RSV pre-F could significantly boost RSV A2 neutralization titers over RSV post-F boost levels (Fig 7), but that neither RSV post-F nor pre-F, with or without adjuvant, could boost T cell responses to levels similar to what was observed in the young seronegative mice (Fig 8). One of the caveats of the seropositive aged mouse model is that prior RSV infection confers complete protection from subsequent viral challenge as evidenced in Fig 3 where all animals, both young and aged, that were intranasally immunized once with live RSV A2 were fully protected in lungs and noses 4 days post re-challenge with RSV A2. This contrasts with human settings in which prior RSV exposure does not provide sufficient protection in many cases. To that extent, further establishment of correlates of protection in older adults, and evaluation of new vaccine candidates in an older adult, human challenge model could be beneficial. Jozwik et al., recently demonstrated in healthy adults that RSV specific tissue resident memory CD8 T cells were essential to provide early recognition and virus elimination post-viral challenge, thus reducing symptoms severity and viral loads [26].

To date, pre-clinical development for RSV vaccines for the elderly population has been mainly restricted to *in vivo* evaluations in young seronegative animals. Our contrasting findings in young and aged mice suggest that future vaccine development for the older population may benefit from pre-clinical testing of vaccine candidates in aged mice (seronegative and seropositive) to identify the most potent formulations before proceeding to the clinic. Additionally, overcoming T cell mediated immunosenescence might require stronger T cell inducers than classic adjuvant formulations and TLR agonists. It might involve including T cell costimulatory molecules as shown by Lee et al. [27], and potentially other routes of administration and/or vaccine platforms.

## Material and methods

### Ethics statement

All procedures were performed in accordance with federal, state and institutional guidelines in an AAALAC-accredited facility and the MedImmune Institutional Animal Care and Use Committee (IACUC) board approved this research under a specified protocol (MI-13-0004), and all animal work was performed in accordance with the IACUC policies. MedImmune is registered with the United States Department of Agriculture (USDA) and applies the standards for the institutional animal care and use program as outlined in the Guide for the Care and Use of Laboratory Animals (Guide), Eighth Edition, National Research Council (NRC), 2011. Animals were lightly anesthetized with isoflurane for immunizations and blood draws, and euthanized with carbon dioxide for terminal organ harvests.

### Vaccine components

The soluble post-fusion (RSV post-F) and pre-fusion (RSV pre-F, DS- Cav1 construct) proteins were expressed, purified and characterized as described in Schneider-Ohrum et al.[23]. GLA-SE [28] was provided by and licensed from Immune Design Corporation (Seattle, WA). GLA-SE was used at 2.5 μg GLA in 2% SE per dose.

### Animals, immunizations and RSV challenge

BALB/c mice at 12 months of age were purchased from Envigo (Dublin, VA) and aged to 18 months of age. The young BALB/c mice were 6–7 weeks of age. The animals were housed under pathogen-free conditions at MedImmune. Animals were lightly anesthetized with isoflurane for immunizations and blood draws, and euthanized with carbon dioxide for terminal organ harvests. For immunogenicity and efficacy studies in RSV seronegative mice, post-fusion and pre-fusion RSV F alone, or RSV F + GLA-SE were administered by intramuscular (i.m.) injection on days 0 and 21. RSV A2 (1 x 10^6^ PFU) (positive control) was administered intranasally (i.n.) on day 0 under isoflurane anesthesia. As a negative control, one group of animals received protein storage buffer [20 mM histidine/histidine-HCl, 23 mM potassium chloride, 7% (weight/volume) sucrose, 0.01% polysorbate 80, pH6.5]. Animals were challenged with RSV A2 (1 x 10^6^ PFU) by i.n. administration on day 35. For studies in RSV seropositive, aged mice, the animals were infected at age 12 months (day 0) with RSV A2 (1 x 10^6^ PFU) and 60 days later with RSV B9320 (1 x 10^6^ PFU). The mice were then immunized i.m. at day 176 (18 months of age) with buffer alone, post-fusion and pre-fusion RSV F alone, or RSV F + GLA-SE. At day 190 the animals were challenged with RSV A2 (1 x 10^6^ PFU).

### Microneutralization assay

Serum samples were heat-inactivated at 56°C for 45 min. In 96-well plates, the positive control antibody (palivizumab) was serially diluted by 3 fold increments (starting at 8 μg/mL) in cell culture media (minimal essential medium (MEM) supplemented with 5% heat-inactivated fetal bovine serum (FBS), 2 mM L-glutamine, 100 U of penicillin/mL, and 100 μg of streptomycin/mL (all from Invitrogen)) for a final volume of 50 μL. In duplicate, the test sera (starting dilution 1:8) were serially diluted by 3-fold increments in cell culture media for a final volume of 50 μL. 25 μL of cell culture media were added to the first dilution to bring the volume back to 50 μL, bringing the starting dilution down to 1:16. Each serum dilution was mixed with 50 μL RSV A2 at 500 PFU per well. Following a 2 h incubation at 37°C with 5% CO_2_, 2.5 × 10^4^ HEp-2 cells in 100 μL volume were added to each well. Cells plus virus and cell only wells served as controls. After 3 days of incubation at 37°C with 5% CO_2_, the cell culture medium was removed and the monolayer was fixed with chilled 80% acetone. RSV replication was visualized by immunostaining with an HRP-labeled 1331H monoclonal antibody [29]. The reciprocal log_2_ of the IC_50_ was determined for each serum sample using Prism GraphPad software (version 7.02). The lower limit of detection (LLOD) of this assay is 4 log_2_. If an IC_50_ value could not be calculated, the log_2_ of the initial dilution (1:16) was used for analysis.

### Serum IgG ELISA

Serological responses were evaluated with sera collected at the day of sacrifice by a standard ELISA on RSV post-F coated (100 ng/well) plates. Control antibodies (purified 1331H for total IgG and IgG2a and purified 1308F for IgG1) [29] were serially diluted by 3-fold increments starting from a concentration of 1 μg/mL in sample diluent (PBS with 1% BSA and 0.05% Tween 20). Samples were diluted in sample diluent at 1:100 for non-immunized animals, at 1:10^5^ and 1:10^6^ for RSV A2-immunized animals, and at 1:10^6^ and 1:10^7^ for RSV sF + adjuvant-immunized animals. Bound total IgG, IgG1 or IgG2a were detected with the appropriate HRP-labeled antibody (goat anti-mouse IgG from Dako, and goat anti-mouse IgG1 and IgG2a antibodies from Jackson ImmunoResearch Laboratories, Inc). The serum antibody titers were calculated based on the standard curves to determine μg/ml of each antibody type (SoftMax Pro 5.4).

### Viral plaque assays

Lungs or nasal turbinates (NT) were collected 4 days post RSV challenge and placed in cold balanced Hanks salt solution supplemented with 1x sucrose phosphate in tissue homogenization tubes (MP Biomedicals) and homogenized using an MP FastPrep24 instrument (MP Biomedicals). The clarified supernatants were serial diluted and placed onto sub-confluent HEp-2 cells in 24-well plates. After a 90 min incubation, supernatants were removed and cells were overlaid with MEM+FBS+Pen/Strep supplemented with 0.75% methylcellulose. After 5 days, cells were fixed with 100% methanol for 15 minutes. Following fixation, cells were blocked for 1 h in 5% nonfat milk and stained for 1 h with a RSV goat polyclonal antibody (Chemicon) followed by staining for 1 h with a HRP-conjugated rabbit anti-goat antibody (DAKO). To visualize plaques, cells were incubated with AEC substrate ready-to-use (DAKO) followed by a water rinsing step. PFU/g were calculated based on number of plaques, dilution factors, and tissue weight.

### IFNγ Elispot

Splenocytes were isolated as previously described [30]. From this preparation, the number of mouse splenocytes secreting gamma interferon (IFNγ) was determined by enzyme-linked immunospot (ELISPOT) assay (BD Biosciences, San Diego, CA) according to the manufacturer’s recommendations. For the in vitro stimulation, splenocytes from individual mice (5×10^5^/well) were incubated with a RSV F specific CD8 peptide (KYKNAVTEL), RSV F specific CD4 peptide (GWYTSVITIELSNIKE) or RSV F overlapping peptide pool at a concentration of 1 μg/ml per peptide (JPT, Berlin, Germany). Controls included splenocytes that were stimulated with Cell Stimulation Cocktail (eBioscience, San Diego, CA) or medium alone stimulated. Following 20 h of incubation in the presence of peptide at 37°C in a humidified incubator, the ELISPOT assay was completed and spots were counted by an ELISPOT assay reader (AID, Germany). For analysis, the spot counts in medium control wells were subtracted from the specific spot count after peptide stimulation, and the difference is reported as the number of spot-forming cells (SFC) per 1 × 10^6^ splenocytes.

### Flow cytometry

Splenocytes from RSV infected mice were harvested 4 days post challenge and isolated as previously described [30]. Lung cells were collected 4 days post challenge and isolated as previously described [31] with the exception that lungs were processed with a gentleMACS dissociator in 5 mL digestion buffer instead of manual dissociation and incubated at 37°C for 30 min.

Eosinophil staining and gating were performed as previously described [32]. Cells were surface-stained with antibodies specific to Ly6c (Clone: HK1.4 Biolegend), Siglec F (Clone: E50-2440 BD Biosciences), Ly6g (Clone: 1A8 Biolegend), MHCII (Clone: M5/114.15.2 Biolegend), CD11c (clone N418, Biolegend), CD11b (Clone: M1/70, eBioscience), F4/80 (Clone: BM8, eBioscience), and CD45 (Clone 30-F11, Biolegend) for 30 min at 4°C and fixed with fixation buffer (eBioscience). 300,000 events were collected on a BD FACS LSR II instrument and analyzed using FlowJo software (Tree Star, Ashland OR).

For intracellular cytokine staining (ICS) cells were incubated with 1 μg/mL of RSV F overlapping peptide pool (JPT, Berlin, Germany) for 12 hours in the presence of 10 μg/mL of brefeldin A (BFA) (BD Biosciences) at 37°C. Following stimulation, cells were blocked with CD16/32 (BD Biosciences) and stained with Fixable Live/Dead dye (ThemoFisher) for 20 min. Cells were then surface stained with antibodies specific to CD4 (Clone: RM4-5, BD Biosciences), CD8 (Clone: 53–6.7, BD Biosciences), and CD 19 (Clone: 6D5, Biolegend) for 20 min. Fixed/Permed with kit (BD Biosciences, #554715) and stained intracellularly for CD3 (Biolegend), IFNγ (Clone: XMG1.2, BD Biosciences), TNFα (Clone: MP6-XT22, BD Biosciences) and IL-2 (Clone: JES6-5H4, BD Biosciences). 500,000 events were collected on a BD FACS LSR II instrument and analyzed using FlowJo software (Tree Star, Ashland OR). The total number of cytokine producing cells was calculated after subtraction of background staining using BFA only controls.

For Pentamer staining, the cells were blocked with CD16/32 (BD biosciences) and stained with Fixable Live/Dead (ThemoFisher) for 20 minutes. Following blocking, cells were surfaced stained with MHCI Pentamer (KYKNAVTEL) (ProImmune), and antibodies specific to CD3 (Clone: 17A2, BD Biosciences), CD4 (Clone: RM4-5, BD Biosciences), CD8 (Clone: 53–6.7, BD Biosciences) and CD19 (Clone 6D5, BD Biosciences) for 30 minutes, and fixed with fixation buffer (eBioscience). Cells were collected on a BD FACS LSR II instrument and analyzed using FlowJo software (Tree Star, Ashland OR).

### Statistical analysis

Sample size estimation was assessed with 80% power to detect differences in treatment groups (based on pilot data) using a two group Satterthwaite t-test with a 0.05 two-sided significance level. Sample size estimation was performed using nQuery + nterim 3.0. Statistical assumptions were assessed prior to analysis. For two-group comparisons, an unpaired t test was applied, including Welch’s correction when group standard deviations were deemed unequal. For comparisons including groups with values at the LLOD, analysis was performed using a Mann Whitney test. When *all* group values were approximately equal to LLOD, a statistical analysis was not performed. Multiplicity-adjusted 2-sided P values are reported. A P value of <0.05 was considered significant. Analyses were performed with GraphPad Prism version 7.02.
